# Supplementary material for: NmeCas9 is an intrinsically high-fidelity genome-editing platform
Source: Genome Biol. 2018 Dec 5;19:214. doi: 10.1186/s13059-018-1591-1 (PMC6282386; doi:10.1186/s13059-018-1591-1)
Supplement: Supplementary file 1 — Figure S1. (A) Sequences of sgRNAs associated with their respective DNA targets used in Fig. 1b. (B) Schematic representation of the split-GFP reporter system used in Figs. 1b, 2a, and 6a. Figure S2. NmeCas9 does not lead to an increase in γH2AX levels in mouse ESC or human HEK293T cells. Figure S3. SpyCas9 and NmeCas9 exhibit similar editing activity when targeting the AAVS1 locus. Figure S4. NmeCas9 and SpyCas9 editing efficiencies at dual target sites (DTSs) that can be cleaved by both enzymes. Figure S5. SITE-Seq read counts and heat map of the frequencies of indels by length at DTS3, DTS7, and DTS8 for SpyCas9 and NmeCas9. Figure S6. Positions of insertions and deletions at the DTS3, DTS7, and DTS8 sites. Figure S7. Sizes of insertions and deletions at the DTS3, DTS7, and DTS8 sites. Figure S8. Fractions of all mutations that are insertions or deletions at the DTS3, DTS7, and DTS8 sites for SpyCas9 and NmeCas9. Figure S9. As in Figure S6. Figure S10. As in Figure S7. Figure S11. GUIDE-seq read counts at NmeCas9 on- and off-target sites. Figure S12. Guide truncation series for Protospacer 9, NTS33 and NTS32. Figure S13. GUIDE-seq analysis to determine whether NmeCas9 sgRNA truncation leads to de novo off-target events. Figure S14. NmeCas9 guide length requirements at the NTS1C and NTS1C-OT1 editing sites. (PDF 5232 kb) [file 13059_2018_1591_MOESM1_ESM.pdf]

Fig. S1

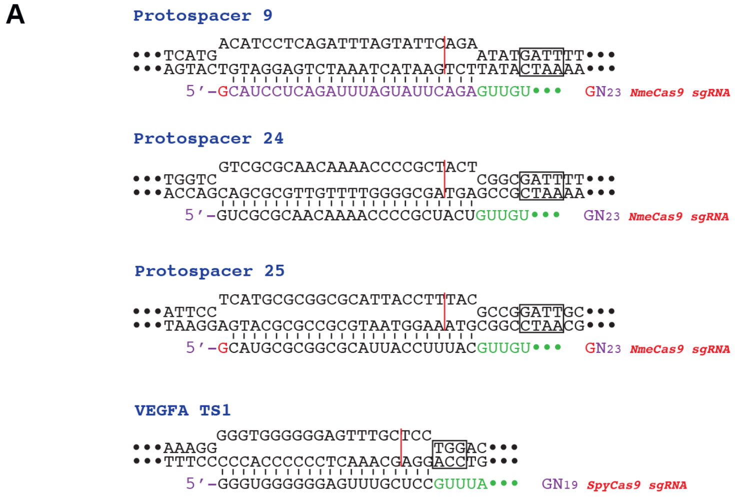

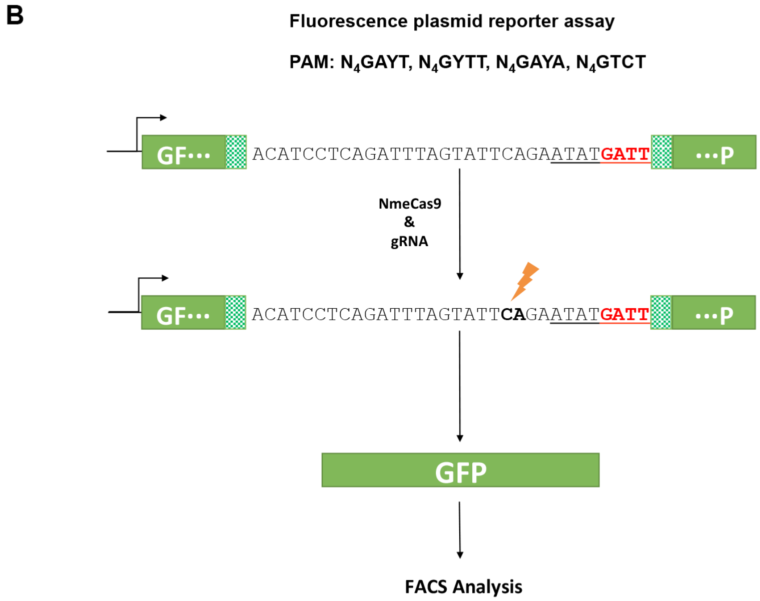

Figure S1. (*A*) Sequences of sgRNAs associated with their respective DNA targets used in Fig. 1B. Black, DNA residues; boxed nts, PAM; red line, NmeCas9 cleavage site; red and purple, mismatched and complementary nts (respectively) in the NmeCas9 sgRNA guide region; green, NmeCas9 sgRNA repeat nts. Numbers on the right represent the length of the guide sequence, with red G(s) depicting a target-mismatched nt and N_x representing the number of complementary nts. (*B*) Schematic representation of the split-GFP reporter system used in Figs. 1B, 2A, and 6A.

Fig. S2

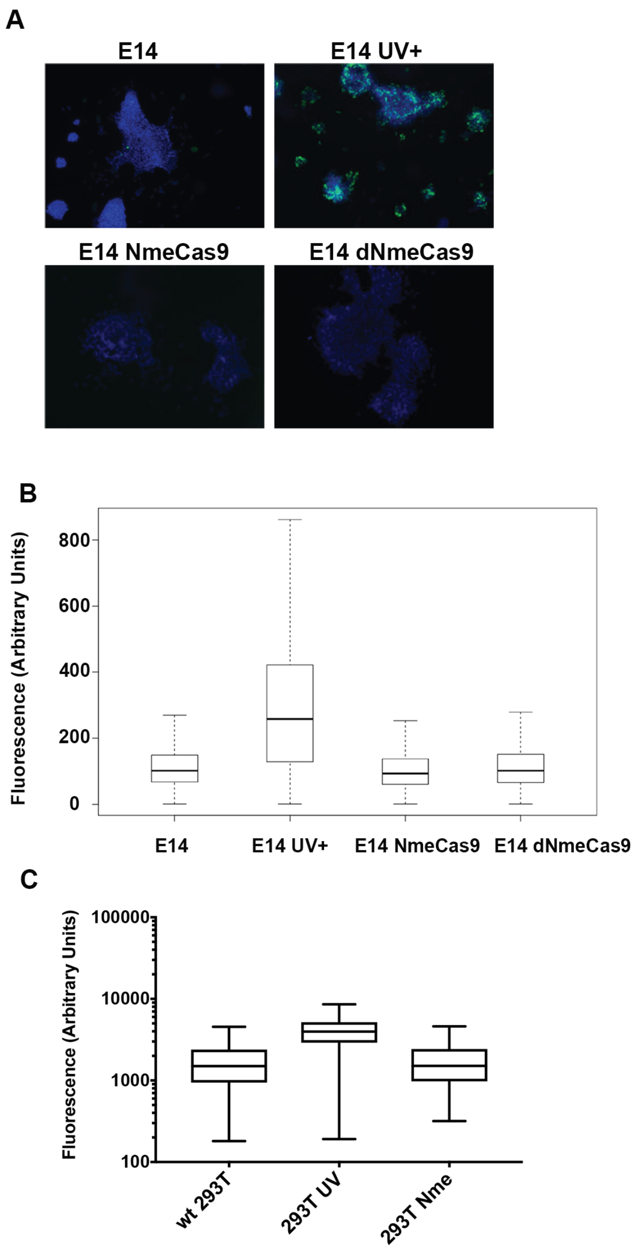

Figure S2. NmeCas9 does not lead to an increase in γH2AX levels in mouse ESC or human HEK293T cells. (*A*) Microscopy of immunoflourescence staining with DAPI (blue) and γH2AX (green). mESCs were crosslinked and stained with DAPI and anti-γH2AX. Cells irradiated with UV (top right) were used as a positive control for γH2AX staining.

(*B*) Box plots of flow-cytometric analysis of γH2AX staining in mESCs. (*C*) Box plots of flow cytometric analysis of γH2AX staining in various HEK293T cells. Inside line represents the median, bottom end of whisker being data of first quartile minus 1.5 times of interquartile range (no less than 0), and top end of whisker being data of third quartile plus 1.5 times of interquartile. All experiments were done in duplicate.

Fig. S3

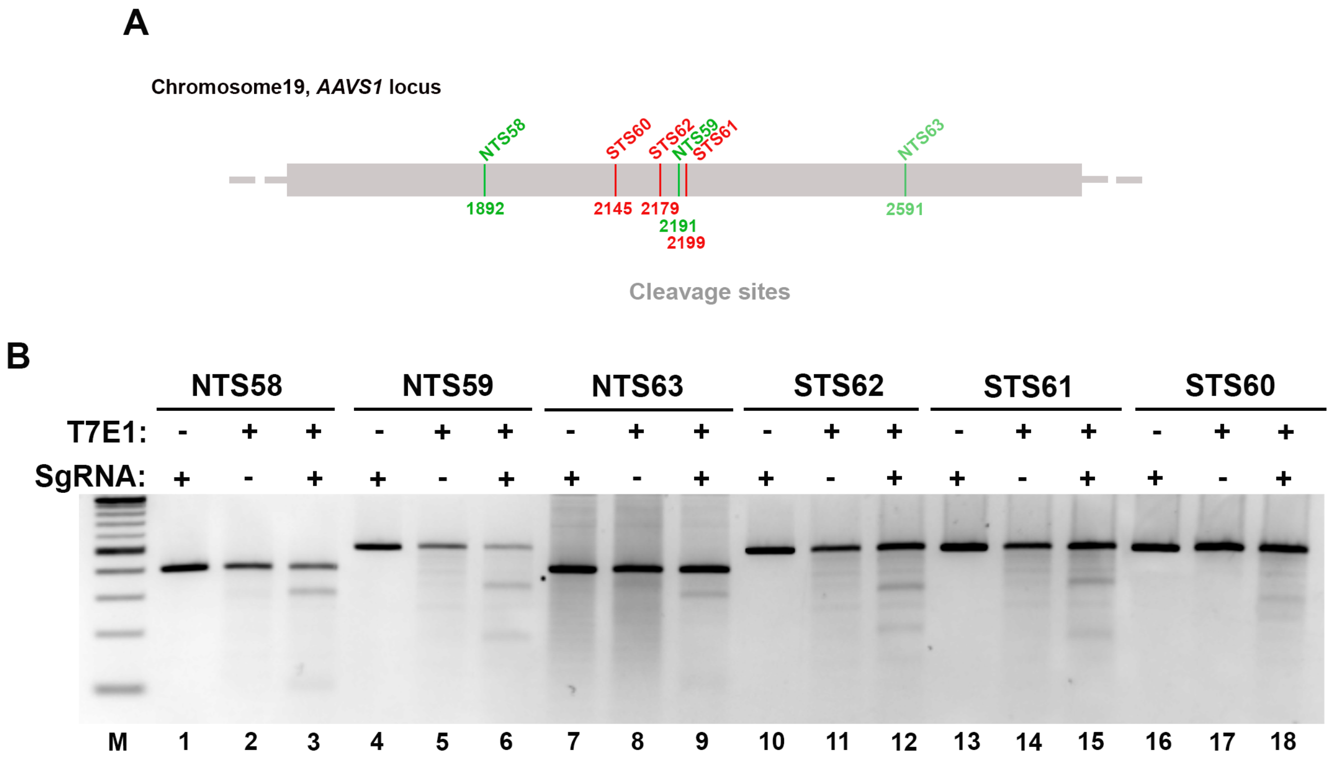

Figure S3. SpyCas9 and NmeCas9 exhibit similar editing activity when targeting the *AAVS1* locus. (*A*) Schematic representation of the *AAVS1* locus showing the relative position of each target site. NTS: NmeCas9 target site (green), STS: SpyCas9 target sites (red). (*B*) T7E1 analyses of NmeCas9 and SpyCas9 editing efficiencies at the target sites depicted in (*A*).

Fig. S4

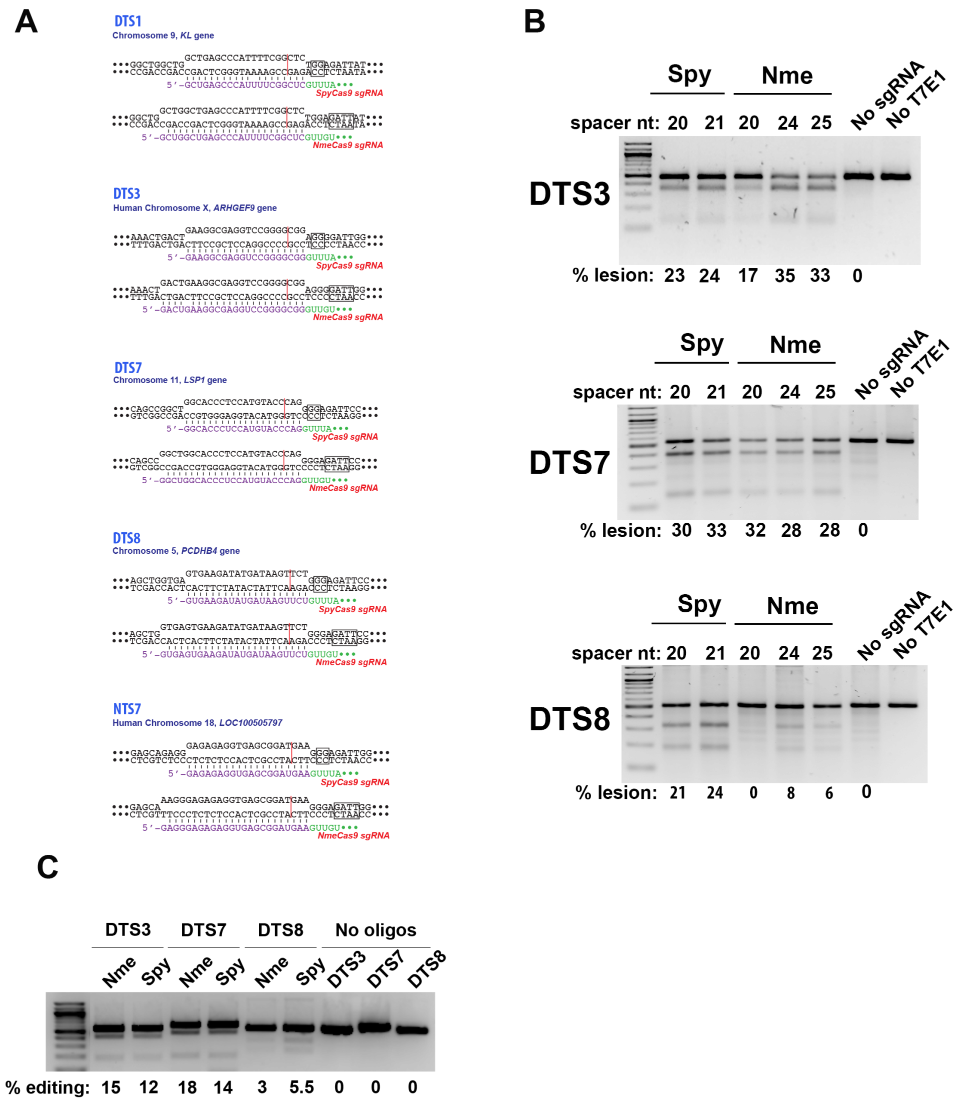

Figure S4. NmeCas9 and SpyCas9 editing efficiencies at dual target sites (DTSs) that can be cleaved by both enzymes. (*A*) Schematic representations of guide-target interactions of NmeCas9 and SpyCas9 sgRNAs at five genomic dual target sites. The DTS1, DTS3, DTS7, and DTS8 guides are perfectly complementary at all guide positions, including the 5'-terminal G residues. For NTS7, the 5'-terminal G of the NmeCas9 sgRNA is wobble-paired to the target. Colors are as in Supplemental Fig. 1. (*B*) T7E1 analysis of SpyCas9 and NmeCas9 editing efficiencies at the DTS3, DTS7, and DTS8 sites. Variations in guide length are indicated at the top, and editing efficiencies are given underneath each lane. (*C*). Restriction endonuclease (*Nde*I) analysis of dsODN incorporation at DTSs during SpyCas9 and NmeCas9 targeting. In the presence of the GUIDE-seq dsODN, a subset of repair events at SpyCas9/NmeCas9 cleavage sites result in dsODN incorporation; these events are reflected by *Nde*I cleavage.

Fig. S5

A

	No Guide	0	0	0	0
**NmeCas9**	NTS1C	2	2	5	9
	NTS3	1	1	2	10
	NTS30	0	0	0	1
	NTS32	1	3	8	34
	NTS33	1	1	1	5
	NTS4B	1	1	1	1
	NTS4C	0	0	1	1
	NTS55	1	1	2	5
	NTS59	0	1	2	4
	NTS7	1	1	1	3
	NTS8	1	1	11	22
	NTS9	1	1	1	1
**SpyCas9**	STS62	379	1473	4988	5783
	RNP concentration (nM):	4	16	64	256

B

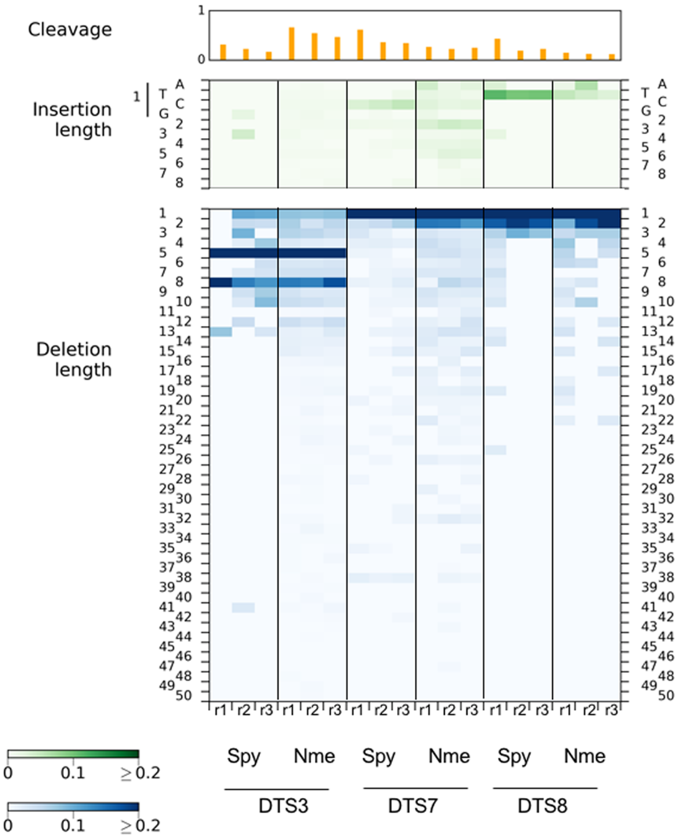

Figure S5. SITE-Seq read counts. (*A*) Number of NmeCas9 sites recovered using SITE-Seq across 4-256 nM Cas9 (n = 1 for each data point; the on-target site is included in this analysis). (*B*) A heat map of the frequencies of indels by length at DTS3, DTS7, and DTS8 for SpyCas9 and NmeCas9 (r1-r3 refers to three replicates). Insertion lengths of 1 to 8 nucleotides are displayed (green). Single-base insertions are separated by nucleotide identity (A, T, C, G). Deletion lengths of 1 to 50 nucleotides are displayed (blue). The color intensity scales with frequency as a fraction of mutant reads up to 0.2. The bar graph above (orange) displays editing efficiency as a fraction of total reads.

Fig. S6

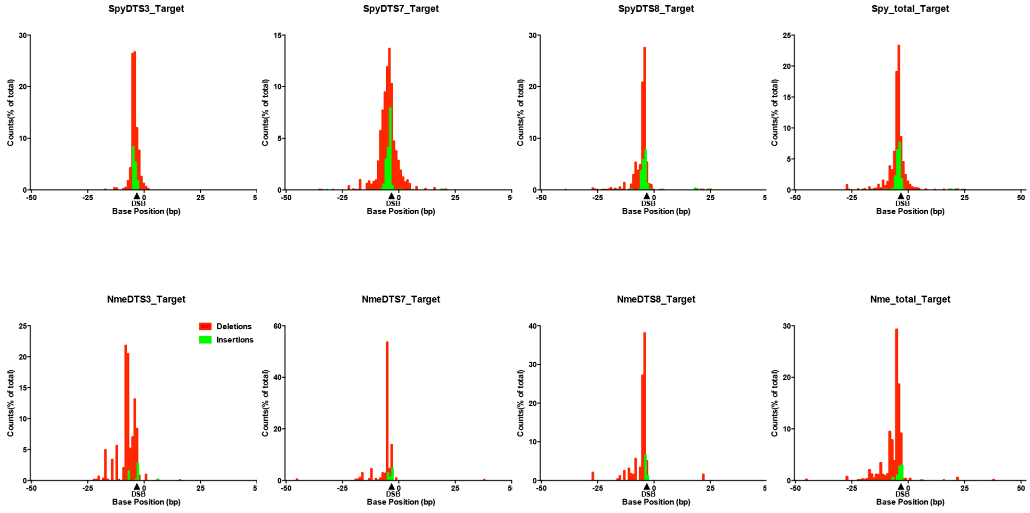

Figure S6. Positions of insertions (green) and deletions (red) at the DTS3, DTS7, and DTS8 sites, or all three sites combined (right), for SpyCas9 (top row) and NmeCas9 (bottom row) editing. See Fig. 4D. Base position (x-axis) bins all deletions or insertion based on the 5'-most base (top strand, relative to the PAM) where the deletion or insertion begins. Counts (y-axis) indicate the fraction of the total indels that are found in each bin. DSB indicates the anticipated position of the double-strand break.

Fig. S7

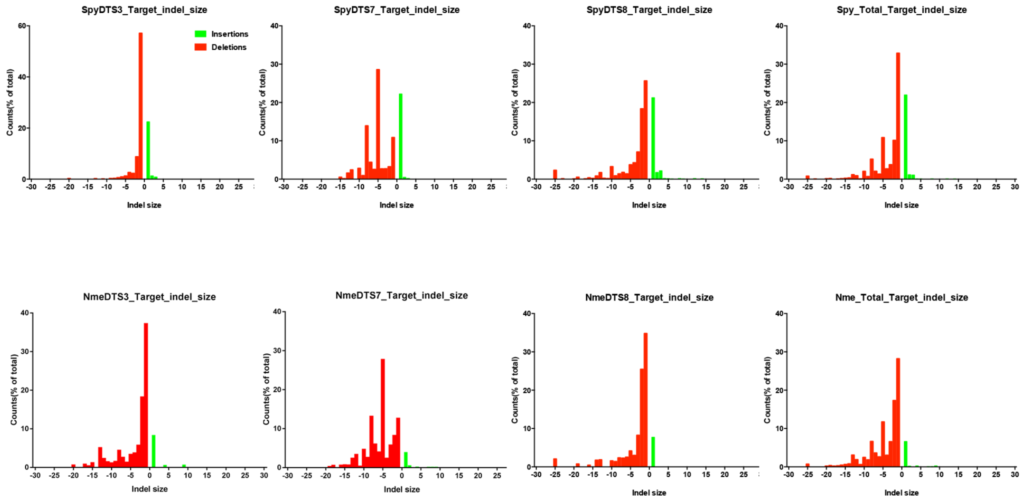

Figure S7. Sizes (in nts) of insertions (green) and deletions (red) at the DTS3, DTS7, and DTS8 sites, or all three sites combined (right), for SpyCas9 (top row) and NmeCas9 (bottom row) editing. See Fig. 4D. Indel size (x-axis) indicates the total number of deleted or inserted nucleotides. Counts (y-axis) indicate the fraction of the total indels that are found in each bin.

Fig. S8

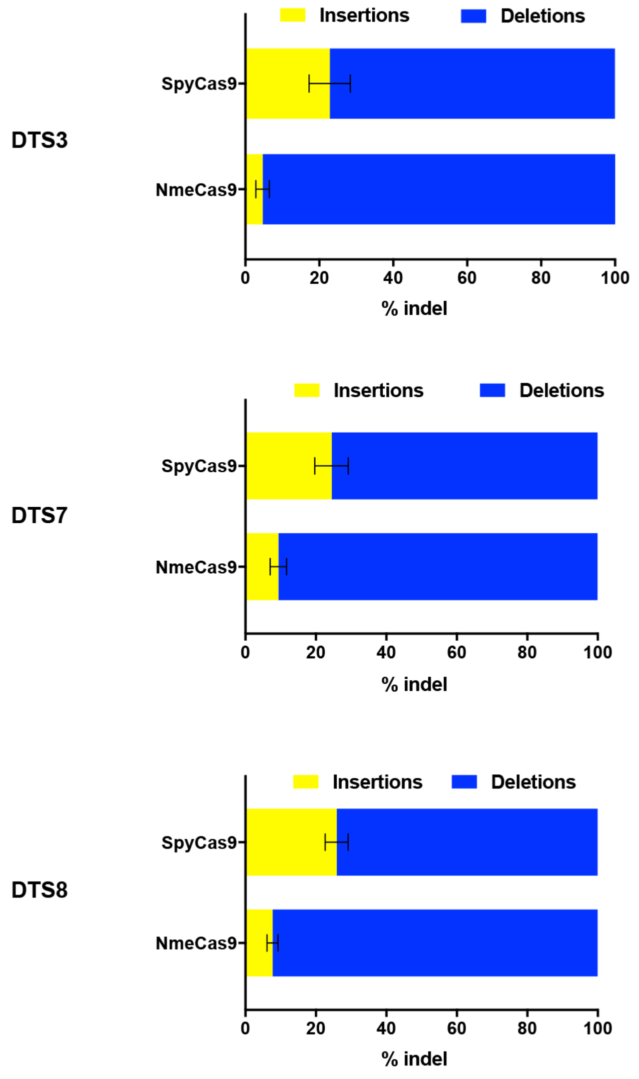

Figure S8. Fractions of all mutations that are insertions (yellow) or deletions (blue) at the DTS3, DTS7, and DTS8 sites for SpyCas9 and NmeCas9, as indicated. Error bars are ± s.e.m. from three biological replicates performed on different days.

Fig. S9

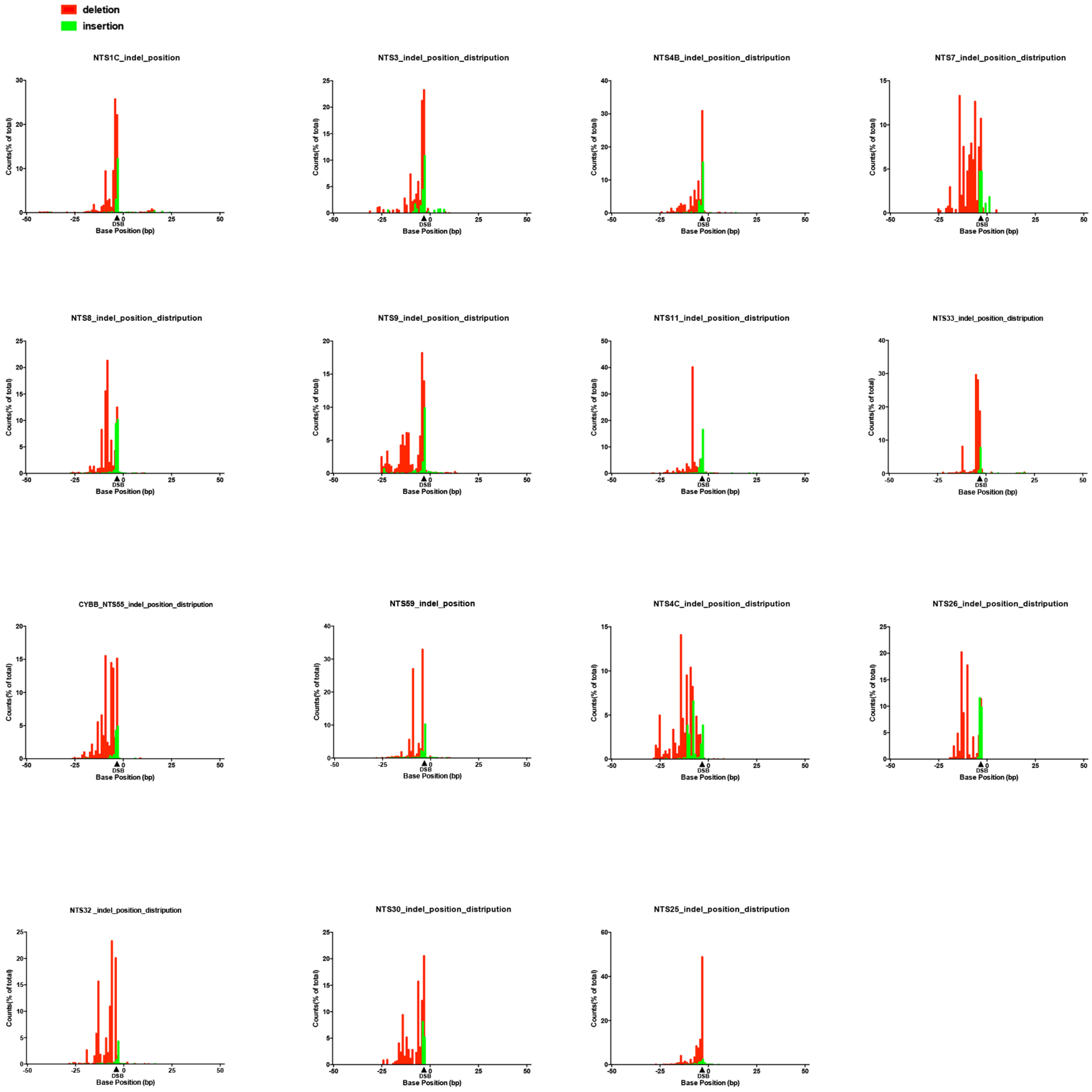

Figure S9. As in Supplemental Fig. 6, but for the guides/targets used in Figs. 1E and 2D. Data for NTS27 is not included in this analysis due to low indel-containing read counts.

Fig. S10

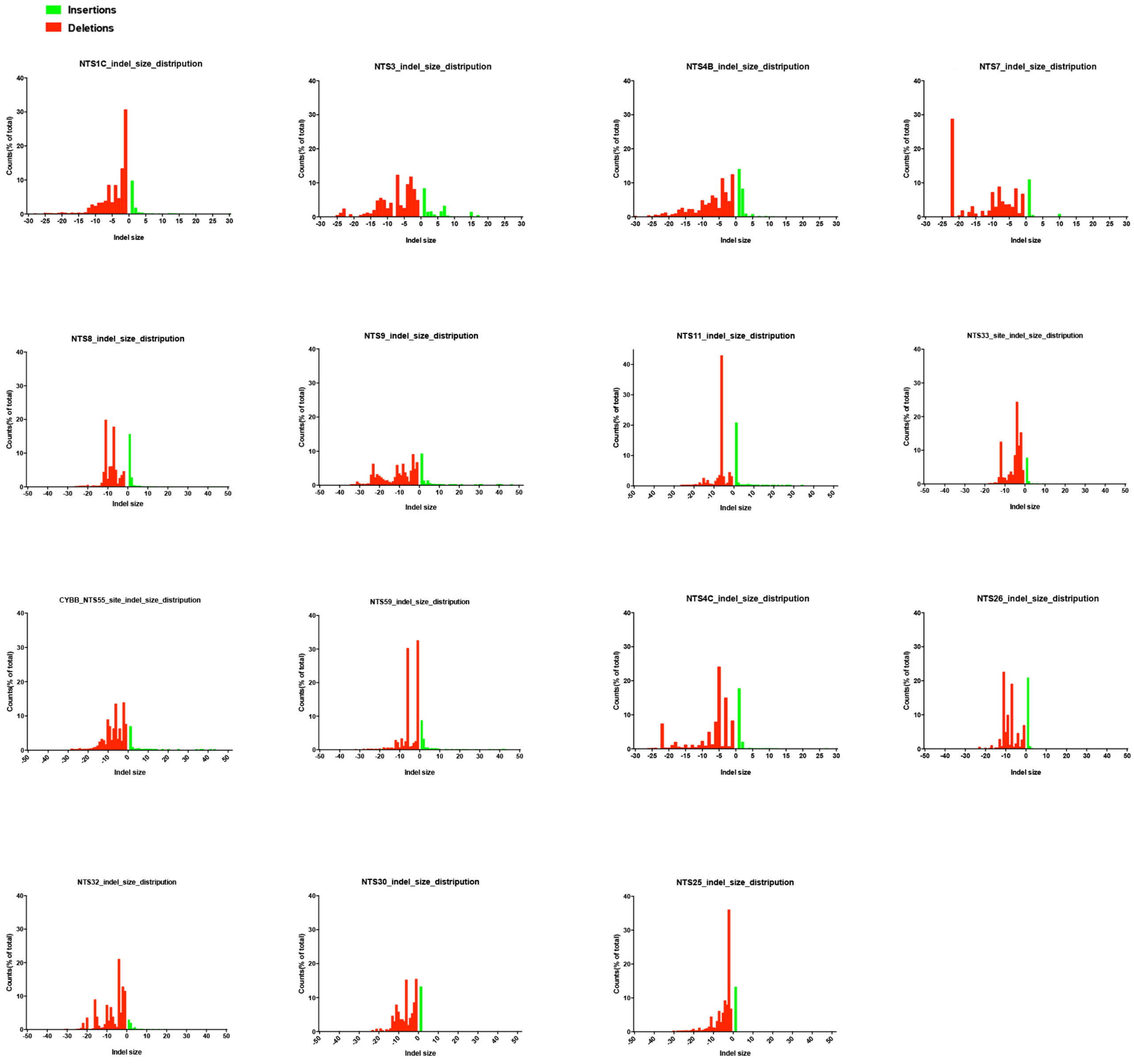

Figure S10. As in Supplemental Fig. 7, but for the guides/targets used in Figs. 1E and 2D. Data for NTS27 is not included in this analysis due to low indel-containing read counts.

Fig. S11

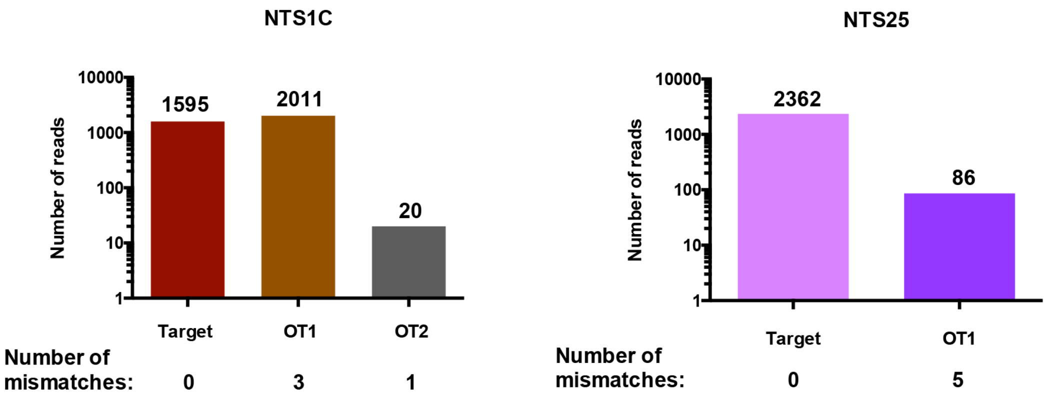

Figure S11. GUIDE-seq read counts at NmeCas9 on- and off-target sites when using sgRNAs targeting NTS1C (left) and NTS25 (right) (see Fig. 5B).

Fig. S12

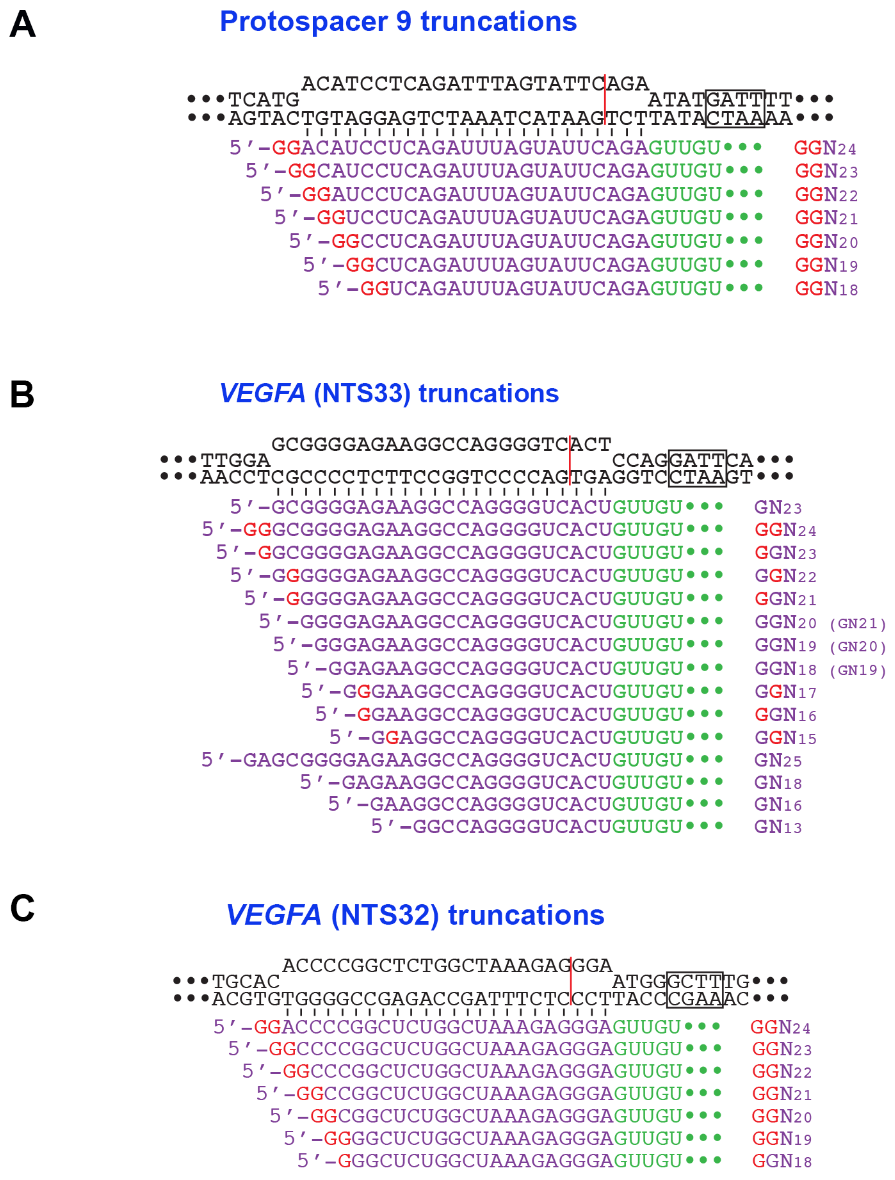

Figure S12. Guide truncation series for (*A*) Protospacer 9 (see Fig. 6A), (*B*) NTS33 (see Fig. 6B-C), and (*C*) NTS32 (see Fig. 6D-E).

Fig. S13

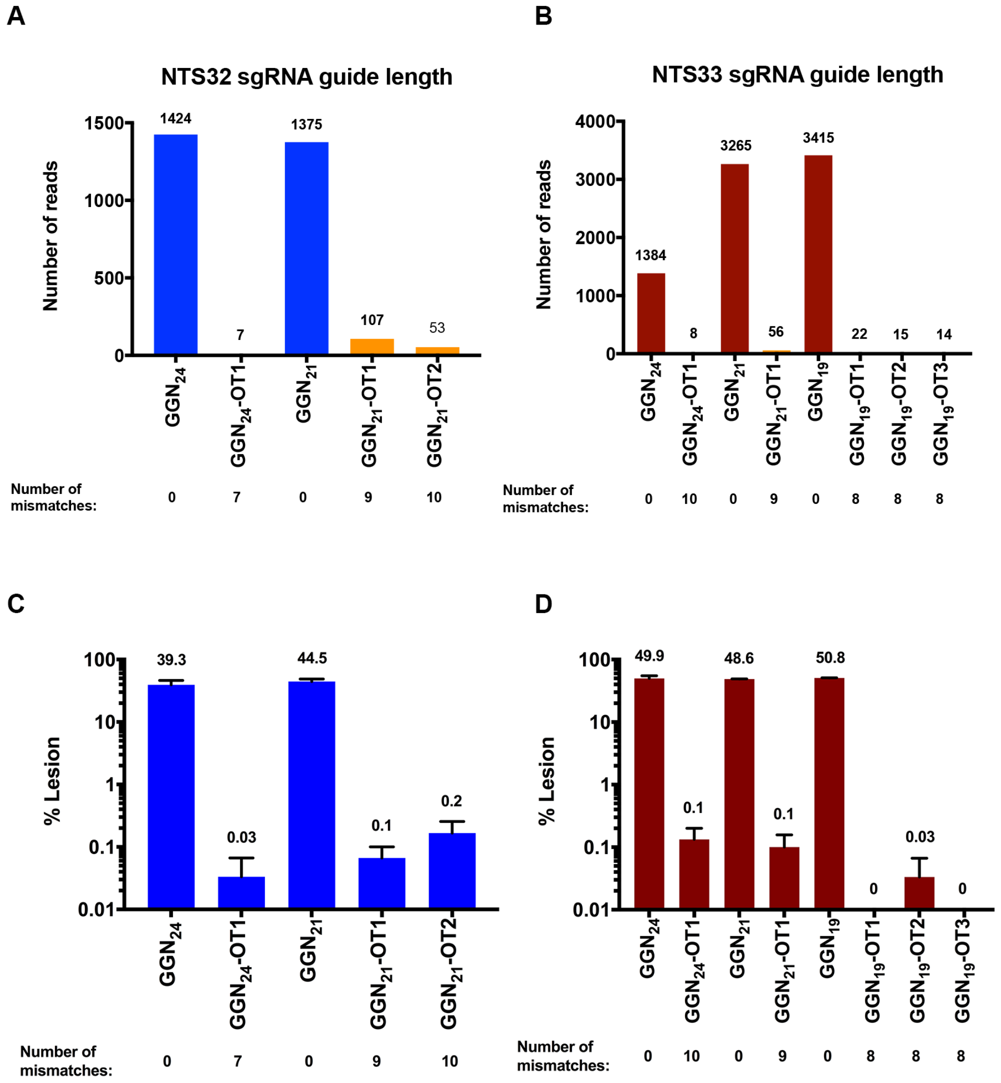

Figure S13. GUIDE-seq analysis to determine whether NmeCas9 sgRNA truncation leads to *de novo* off-target events. (*A*) GUIDE-seq read counts at on-target (blue) and off-target (orange) sites with NTS32 sgRNAs with 24 and 21 nts of target complementarity. The Bioconductor package GUIDEseq version 1.1.17 (Zhu et al., 2017) was used with default setting except that PAM.size = 8, PAM = "NNNNGATT", min.reads = 2, max.mismatch = 10, allowed.mismatch.PAM = 4, PAM.pattern = "NNNNNNNN$", BSgenomeName = Hsapiens, txdb = TxDb.Hsapiens.UCSC.hg19.knownGene, orgAnn = org.Hs.egSYMBOL gRNA.size was set to length of the gRNA used. Analysis on truncated sgRNAs with 24 to 16 nucleotides complementarity to genomic target site (*A*) NTS32 and (*B*) NTS33. Targeted deep-sequencing validation of potential *de novo* off-target sites of (*C*) NTS32 and (*D*) NTS33.

Fig. S14

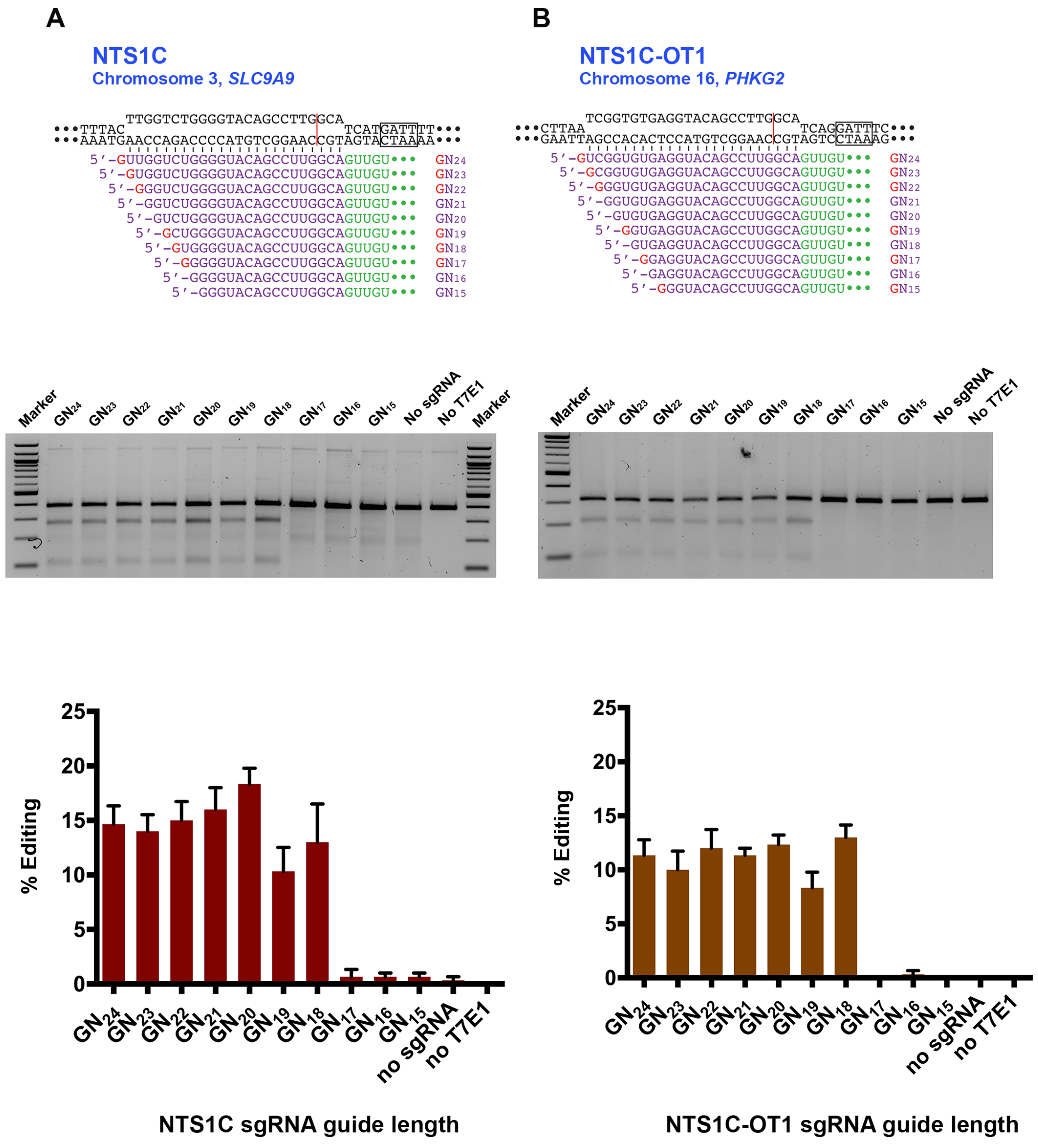

Figure S14. NmeCas9 guide length requirements at the NTS1C and NTS1C-OT1 editing sites. (*A*) Schematic depiction of full-length and truncated sgRNAs complementary to NTS1C (top), T7E1 analyses of on-target editing (middle), and quantitation of the T7E1 data (bottom). The bar graphs represent mean values ± s.e.m. from three biological replicates performed on different days. (*B*) As in (*A*), but for the sgRNA series complementary to NTS1C-OT1. See Fig. 7.
